# Association between intraoperative dexmedetomidine and survival outcomes after colorectal cancer surgery: a retrospective cohort study

**DOI:** 10.3389/fonc.2025.1693496

**Published:** 2025-12-02

**Authors:** Shirong Chen, Lu Gan, Ruosi Zhang, Xiang Huang, Pei Li, Jiawei Ni, Kexuan Liu, Huamin Liu, Cai Li

**Affiliations:** Department of Anesthesiology, Nanfang Hospital, Southern Medical University, Guangzhou, China

**Keywords:** dexmedetomidine, colorectal cancer, postoperative outcomes, all-cause death, tumor recurrence

## Abstract

**Background:**

The impact of dexmedetomidine (DEX) on postoperative survival outcomes in cancer remains controversial. Our study aimed to investigate the influence of intraoperative DEX administration on postoperative mortality outcomes in colorectal cancer patients.

**Methods:**

This was a retrospective cohort study of adult patients undergoing colorectal cancer surgery in a large academic hospital in southern China between 2011 and 2018. Patients were divided into two groups: the DEX group, in which patients received intravenous DEX during the operation, and the non-DEX group. The primary endpoint was overall death or tumor recurrence, deriving two outcome variables: “all-cause death” and “recurrence or death.” Secondary endpoints included total hospital stay, postoperative hospital stay, and postoperative complications. Multivariable Cox regression and propensity score matching were used to control confounders.

**Results:**

A total of 1,367 adult patients were included, of which 485 pairs were matched. Patients who received intraoperative DEX had a lower all-cause death rate (8.0% *vs*. 14.2%, *P* = 0.002) and a lower recurrence or death rate (14.8% *vs*. 23.1%, *P* = 0.001). Intraoperative DEX administration was associated with a lower risk of all-cause death postoperatively [adjusted hazard ratio (HR) and 95% confidence interval (CI): 0.74, 0.52–1.07 in overall patients; 0.66, 0.45–0.98 in matched patients] compared with non-DEX. The risk of recurrence or death was lower with a marginal significance (HR and 95% CI: 0.75, 0.56–1.01) in matched patients. The total hospital stay and postoperative hospital stay were lower in patients who used DEX than those who did not use it (*β* and 95% CI: −0.96 [−1.71, −0.22] and −0.95 [−1.47, −0.43], respectively, in matched patients). Meanwhile, the risk of postoperative complications was not associated with DEX.

**Conclusions:**

In patients undergoing colorectal cancer surgery, intraoperative DEX administration was associated with better postoperative survival.

## Introduction

With the increasing aging population, the incidence of cancer has risen rapidly. According to global cancer statistics, the number of new colorectal cancer cases exceeded 1.9 million in 2020, with an estimated 940,000 deaths, making it the second leading cause of cancer-related deaths (1). Surgery is the main treatment for patients with non-metastasized colorectal cancer (2), but the use of anesthesia drugs during the intraoperative period may affect the metastasis and spread of cancer, thereby potentially impacting its survival outcomes (3, 4). Therefore, it is urgent to explore the appropriate anesthesia agents to improve postoperative survival outcomes.

Dexmedetomidine (DEX), a highly selective α2-adrenoceptor agonist with anti-sympathetic, anxiolytic, and sedative properties (5), has been utilized in intensive care units and is now widely used as a relatively new anesthetic by anesthesiologists (6). Its use in patients with cancer surgery remains controversial due to conflicting findings from various studies. Studies have shown that DEX can reduce antitumor immunity, promote tumor growth and metastasis (7–9), and even decrease the postoperative overall survival in patients who underwent lung cancer surgery (10). Conversely, studies have suggested that DEX could inhibit the growth of various cancer cells, such as ovarian cancer cells, lung adenocarcinoma cells, and esophageal cancer cells, which might help improve postoperative outcomes (11–13). Moreover, a clinical study has shown that DEX can alleviate postoperative immunosuppression and improve postoperative outcomes in oral cancer patients (14).

Based on these controversial reports, DEX may have opposite postoperative outcomes for different cancers. We hypothesized that DEX might improve postoperative survival outcomes in patients with colorectal cancer because of its antitumor effects, reduced stress and inflammatory response, and protection of immune function (15, 16). Therefore, the aim of our study was to investigate the influence of intraoperative DEX administration on postoperative survival outcomes in colorectal cancer patients.

## Methods

### Study design and participants

**Figure 1:**
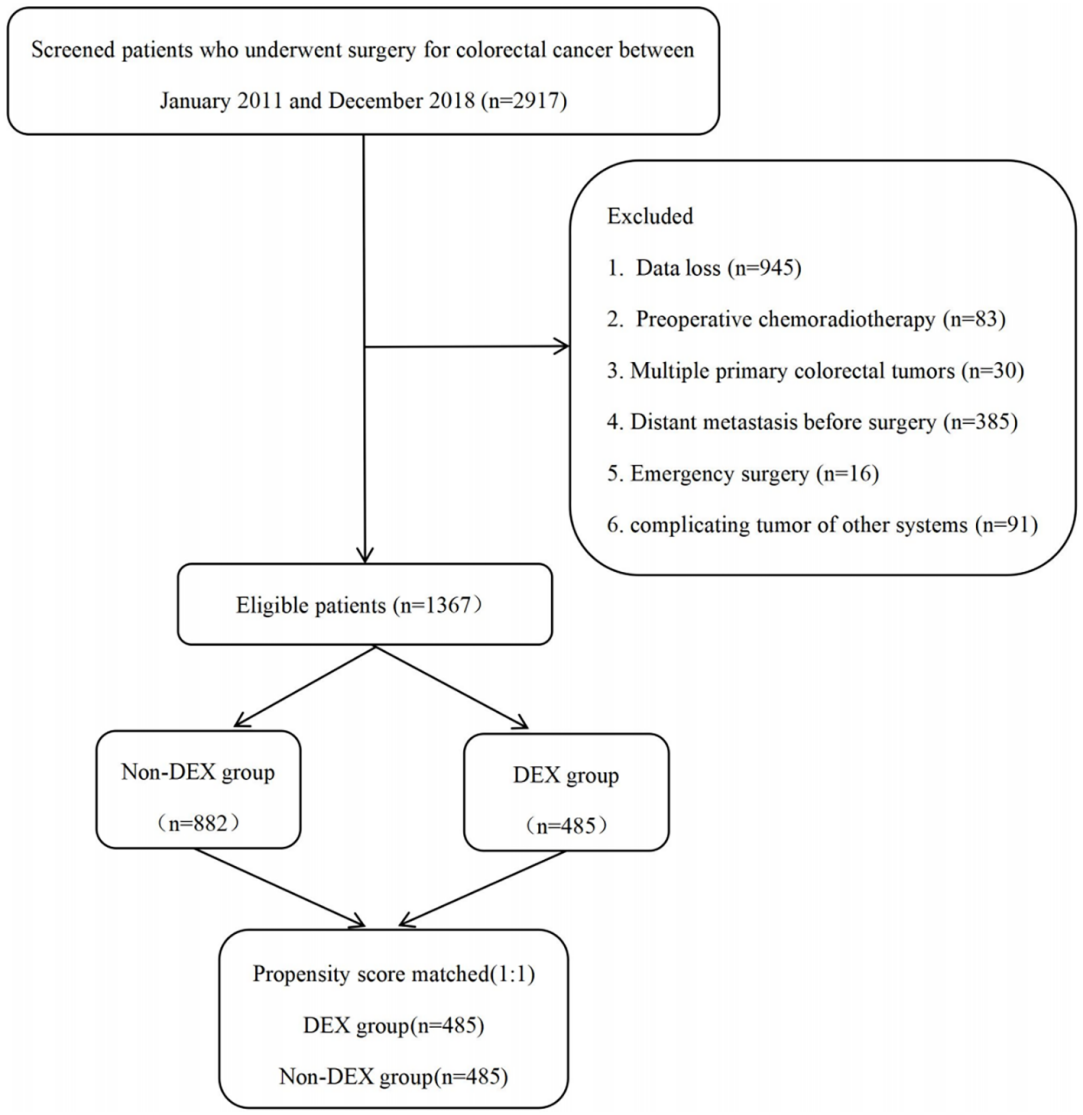
Flowchart of the patient selection.

This was a retrospective analysis from a large tertiary academic hospital in southern China. After obtaining approval from the Ethics Committee of Nanfang Hospital of Southern Medical University (NFEC-2020-050, 24 March 2020), the study cohort included patients who underwent colorectal cancer surgery between January 2011 and December 2018 at Nanfang Hospital of Southern Medical University. Adult patients who underwent colorectal cancer surgery under general anesthesia were included in the analysis, and all patients were staged according to the American Joint Committee on Cancer (AJCC) 7th edition manual for colorectal cancer. All anesthetics used by the patients included inhaled anesthetics, intravenous anesthetics, opioids, and muscle relaxants with non-depolarizing agents. The patient selection flowchart is presented in **Figure 1**. The exclusion criteria included the following: 1) data loss (*n* = 945); 2) preoperative chemoradiotherapy (*n* = 83); 3) presence of multiple primary colorectal tumors (*n* = 30); 4) distant metastasis before surgery (*n* = 385); 5) emergency surgery due to conditions such as intestinal obstruction, bleeding, or perforation (*n* = 16); and 6) complicating tumor of other systems (*n* = 91). Finally, 1,367 patients were included, of which propensity score matching (PSM) acquired 485 pairs.

### Clinical data collection

Clinical data were collected using Perioperative Data Warehouse from the Department of Anesthesiolgy, Nanfang Hospital, Southern Medical University, including age, gender, body mass index (BMI), preoperative hemoglobin (HGB), preoperative albumin (ALB), American Society of Anesthesiologists (ASA) grade, comorbidities, preoperative carcinoembryonic antigen (CEA) level, tumor location, tumor differentiation, pathological tumor node metastasis (TNM) stage, surgical type, previous abdominal surgery, the duration of operation, intraoperative blood loss, total intraoperative infusion volume and blood transfusion, postoperative chemotherapy, postoperative ICU (intensive care unit admission), total hospital stay, postoperative hospital stay, and postoperative complications. We also collected information on the total amount of DEX administered perioperatively. DEX exposure was defined as continuous intravenous infusion initiated after anesthesia induction and before the end of surgery. The infusion rate ranged from 0.2 to 0.7 μg/kg/h, with a median total dose of 60 μg. Patients who did not receive DEX during this intraoperative period were assigned to the non-DEX group.

### Long-term follow-up and outcomes

Long-term follow-up of postoperative mortality and tumor recurrence was performed by investigators who had no knowledge of group assignment and who had been trained for follow-up data collection. Telephone follow-up was utilized to access patient survival information. The primary endpoint of this study was the overall death or tumor recurrence following colorectal cancer surgery, deriving two outcome variables: “all-cause death” and “recurrence or death.” Secondary outcomes included total hospital stay, postoperative hospital stay, and postoperative complications, including wound infection, anastomotic leakage, pulmonary infections, and complications related to urinary retention or bacteremia. Survival time was defined as the duration from the day of colorectal cancer surgery until death from any cause following surgery during follow-up. Disease-free survival time referred to the period from the day of colorectal cancer surgery until either tumor recurrence or death from any cause during the follow-up. Tumor recurrence was confirmed by radiological or pathological evidence.

### Statistical analysis

The normality of continuous variables was assessed using the Kolmogorov–Smirnov test. Continuous variables with a normal distribution were expressed as mean ± standard deviation and compared using the Student’s *t*-test. Continuous variables with a non-normal distribution were expressed as median (interquartile range) and compared using the Mann–Whitney *U* test. Categorical variables were presented as frequencies with percentages and compared using the *χ*² test or continuity-corrected *χ*² test.

Cox proportional hazard regression analyses with hazard ratio (HR) and 95% CI were used to estimate mortality risk. Survival proportion was compared using the Kaplan–Meier method with the log-rank test. Covariates for the multivariable Cox models were selected based on clinical relevance and prior published studies. Preoperative factors were included as confounders, as they influence the exposure factor but are not affected by it. Since DEX was used immediately after anesthesia induction, the intraoperative variables could not affect the exposure factor; thus, these variables were not suitable as confounders. Therefore, the confounder factors in this study included age, sex, BMI, preoperative HGB, preoperative ALB, ASA grade, history of abdominal operation, preoperative CEA, surgery type (open surgery or laparoscopic surgery), TNM stage, differentiation degree, tumor location, and preoperative comorbidity. The Schoenfeld residual method was used to test the proportional hazards assumption, and no significant violations were detected (**Supplementary Table S1**). Two models were applied: model 1 was unadjusted, while model 2 was adjusted for the above confounder factors, in which the comorbidities included hypertension, diabetes, pulmonary disease, and cardiovascular disease.

Propensity score matching analysis with a 1:1 ratio was used to verify the robustness of the results. The logistic regression model was used to calculate the propensity score, with DEX as the dependent variable and all confounder factors mentioned above as matching variables. Propensity score matching was performed using a nearest-neighbor algorithm without replacement, with a caliper value of 0.2 SD of the logit of the propensity score. In the matched cohort, 485 patients were selected for each group. The patients in the DEX and non-DEX groups demonstrated improved homogeneity after matching (**Supplementary Figures S1**, **S2**). A love plot with standard mean difference (SMD) was used to evaluate the balance between matched groups, with SMD <0.1 indicating balance (**Supplementary Figure S3**).

The association of DEX with total hospital stay and postoperative hospital stay was assessed using multiple linear regression with coefficients (*β*) and 95% CIs. The hypothesis test that satisfied the assumptions of linear regression was performed (**Supplementary Tables S2**, **S3**; **Supplementary Figures S4**-**S7**). A logistic regression model with odds ratios (ORs) and 95% CIs was used to determine the association of DEX with postoperative complications. All statistical analyses were performed using R software (version 4.2.3, R Foundation for Statistical Computing, Vienna, Austria). *P*-values <0.05 for the two-sided test were considered statistically significant.

## Results

### Characteristics of patients

Among the 1,367 participants, 485 patients received DEX with a median intraoperative consumption of 60 μg. Patients in the DEX group were younger, had lower ASA grades and worse tumor differentiation, and received more intraoperative infusion volume than those who did not receive DEX. After propensity score matching, an imbalance remained in the intraoperative infusion volume (*P* = 0.004), while other covariates were balanced (**Table 1**).

**Table 1:** Characteristics of patients before and after matching.

Characteristics	Unmatched			Matched		
	DEX	Non-DEX	*P*	DEX	Non–DEX	*P*
*N*	485	882		485	485	
Age, years	57.0 (47.0–64.0)	60.0 (49.0–68.0)	0.001	57.0 (47.0–65.0)	57.0 (47.0–64.0)	0.954
Male, *n* (%)	306 (63.1)	584 (66.2)	0.247	306 (63.1)	321 (66.2)	0.314
BMI, kg/m²	22.7 ± 3.3	22.6 ± 3.2	0.936	22.6 (20.6–24.8)	22.7 (20.5–25.0)	0.969
Preoperative HGB, g/L	123.2 ± 21.3	123.3 ± 20.9	0.936	125.0 (112.0–139.0)	125.0 (111.0–140.0)	0.909
Preoperative ALB, g/L	38.39 ± 4.03	38.21 ± 4.20	0.445	38.4 (35.9–40.9)	38.4 (35.6–41.1)	0.882
ASA grade, *n* (%)			0.001			0.838
I	126 (26.0)	184 (20.9)		126 (26.0)	118 (24.3)	
II	345 (71.1)	633 (71.8)		345 (71.1)	353 (72.8)	
III	14 (2.9)	65 (7.4)		14 (2.9)	14 (2.9)	
Comorbidity, *n* (%)						
Hypertension	93 (19.2)	192 (21.8)	0.259	93 (19.2)	94 (19.4)	0.935
Diabetes	46 (9.5)	92 (10.4)	0.578	46 (9.5)	53 (10.9)	0.458
Cardiovascular disease	36 (7.4)	49 (5.6)	0.171	36 (7.4)	32 (6.6)	0.615
Pulmonary disease	16 (3.3)	28 (3.2)	0.901	16 (3.3)	14 (2.9)	0.711
CEA (ng/mL) > 5, *n* (%)	110 (22.7)	223 (23.5)	0.741	110 (22.7)	114 (23.5)	0.761
Tumor location, *n* (%)			0.646			0.899
Right colon	118 (24.3)	214 (24.3)		118 (24.3)	115 (23.7)	
Left colon	182 (37.5)	311 (35.3)		182 (37.5)	178 (36.7)	
Rectum	185 (38.1)	357 (40.5)		185 (38.1)	192 (39.6)	
Tumor differentiation, *n* (%)			0.024			0.934
Poorly	59 (12.2)	89 (10.1)		59 (12.2)	60 (12.4)	
Moderately	388 (80.0)	684 (77.6)		388 (80.0)	390 (80.4)	
Well	38 (7.8)	109 (12.4)		38 (7.8)	35 (7.2)	
Pathologic TNM stage, *n* (%)			0.821			0.618
0–I	68 (14.0)	114 (12.9)		68 (14.0)	60 (12.4)	
II	214 (44.1)	388 (44.0)		214 (44.1)	209 (43.1)	
III	203 (41.9)	380 (43.4)		203 (41.9)	216 (44.5)	
Surgical type, *n* (%)			0.153			0.867
Open surgery	18 (3.7)	48 (5.4)		18 (3.7)	24 (4.9)	
Laparoscopic surgery	467 (96.3)	834 (94.6)		467 (96.3)	461 (95.1)	
History of abdominal surgery, *n* (%)	25 (5.2)	45 (5.1)	0.966	25 (5.2)	26 (5.4)	0.886
Duration of operation ≥180, min, *n* (%)	118 (24.3)	193 (21.9)	0.302	118 (24.3)	122 (25.2)	0.706
Intraoperative blood loss, mL	50 (30–100)	50 (30–100)	0.001	50 (30–100)	50.0 (30.0–100.0)	0.688
Total intraoperative infusion volume, mL	2,200 (1,850–2,700)	2,000 (1,600–2,200)	<0.001	2,100 (1,700–2,600)	2,200 (1,850–2,700)	0.004
Blood transfusion, *n* (%)	35 (7.2)	64 (7.3)	0.978	35 (7.2)	28 (5.8)	0.362
Postoperative chemotherapy, *n* (%)	274 (56.5)	525 (59.5)	0.277	274 (56.5)	283 (58.4)	0.559
Postoperative ICU, *n* (%)	20 (4.1)	57 (6.5)	0.073	20 (4.1)	24 (4.9)	0.537

DEX, dexmedetomidine; BMI, body mass index; HGB, hemoglobin; ALB, albumin; ASA, American Society of Anesthesiologists; CEA, carcinoembryonic antigen; TNM, tumor node metastasis; ICU, intensive care unit.

### The incidence of outcomes between patients in the DEX and non-DEX groups

Overall, patients in the DEX group had significantly lower incidence rates of all-cause death (8.0% *vs*. 14.9%) and recurrence or death (14.8% *vs*. 22.6%) than the non-DEX group. The incidence rates of all-cause death (8.0% *vs*. 14.2%) and recurrence or death (14.8% *vs*. 23.1%) were also lower in the DEX group after matching. Furthermore, the total hospital stay (13.1 *vs*. 14.0 days) and postoperative hospital stay (6.9 *vs*. 7.9 days) were significantly shorter for patients who received DEX compared to those who did not, with these differences remaining significant after matching (13.1 *vs*. 14.0 days for total hospital stay, 6.9 *vs*. 7.8 days for postoperative hospital stay). No significant difference was observed in the incidence of postoperative complications (**Table 2**).

**Table 2:** The incidence of postoperative outcomes before and after matching.

Variables	Unmatched			Matched		
	DEX	Non-DEX	*P*	DEX	Non-DEX	*P*
Primary outcomes						
All-cause death, *n* (%)	39 (8.0)	131 (14.9)	<0.001	39 (8.0)	69 (14.2)	0.002
Recurrence or death, *n* (%)	72 (14.8)	199 (22.6)	0.001	72 (14.8)	112 (23.1)	0.001
Secondary outcomes						
Total hospital stay, days	13.1 (10.8–17.0)	14.0 (11.7–17.0)	0.001	13.1 (10.8–17.0)	14.0 (11.1–17.0)	0.012
Postoperative hospital stay, days	6.9 (5.8–8.8)	7.9 (6.7–9.7)	<0.001	6.9 (5.8–8.8)	7.8 (6.7–9.0)	<0.001
Postoperative complications, *n* (%)	42 (8.7)	81 (9.2)	0.746	42 (8.7)	41 (8.5)	0.909
Wound infection	7 (1.4)	8 (0.9)	0.362	7 (1.4)	4 (0.8)	0.363
Anastomosis leakage	9 (1.9)	17 (1.9)	0.926	9 (1.9)	8 (1.6)	0.807
Pulmonary infections	10 (2.1)	23 (2.6)	0.529	10 (2.1)	12 (2.1)	0.666
Anastomitis	4 (0.8)	9 (1.0)	0.721	4 (0.8)	1 (0.2)	0.370^a^
Anastomotic ulcers	2 (0.4)	4 (0.5)	1.000	2 (0.4)	1 (0.2)	1.000^a^
Anastomotic stenosis	1 (0.2)	6 (0.7)	0.436^a^	1 (0.2)	4 (0.8)	0.370^a^
Urinary retention	4 (0.8)	1 (0.1)	0.106	4 (0.8)	1 (0.2)	0.370^a^
Bacteremia	9 (1.9)	15 (1.7)	0.835	9 (1.9)	10 (2.1)	0.817
Other	8 (1.6)	14 (1.6)	0.930	8 (1.6)	10 (2.1)	0.634

DEX, dexmedetomidine.

a Continuity correction *χ*^2^ test.

### The associations of DEX with the main outcomes

Cox proportional hazards regression analyses revealed that patients receiving DEX had a lower all-cause death risk prior to matching, although this association only reached marginal significance [adjusted HR and 95% CI: 0.74 (0.52–1.07)]. In the matched cohort, the risk of all-cause death was significantly reduced in patients who received DEX compared to those who did not receive it [adjusted HR and 95% CI: 0.66 (0.45–0.98)]. However, no significant difference was observed in the risk of recurrence or death [adjusted HR and 95% CI: 0.88 (0.67–1.16)] for the prematched cohort, while a marginal decrease in the risk of recurrence or death was observed in the matched cohort [adjusted HR and 95% CI: 0.75 (0.56–1.01)] (**Table 3**).

**Table 3:** The associations of DEX with main outcomes.

	Unmatched [HR (95% CI)]		Matched [HR (95% CI)]
	Model 1	Model 2	Model 3
All-cause death			
Non-DEX	1	1	1
DEX	0.70 (0.49–1.00)	0.74 (0.52–1.07)	0.66 (0.45–0.98)
Recurrence or death			
Non-DEX	1	1	1
DEX	0.86 (0.65–1.12)	0.88 (0.67–1.16)	0.75 (0.56–1.01)

Model 1: unadjusted in overall cohort; model 2: adjusted for age, sex, BMI, TNM stage, preoperative ALB, preoperative HGB, preoperative CEA, differentiation degree, history of abdominal operation, and preoperative comorbidity in the overall cohort; model 3: the matched cohort.

DEX, dexmedetomidine; HR, hazard ratio; CI, confidence interval.

*P < 0.05.

**Figure 2:**
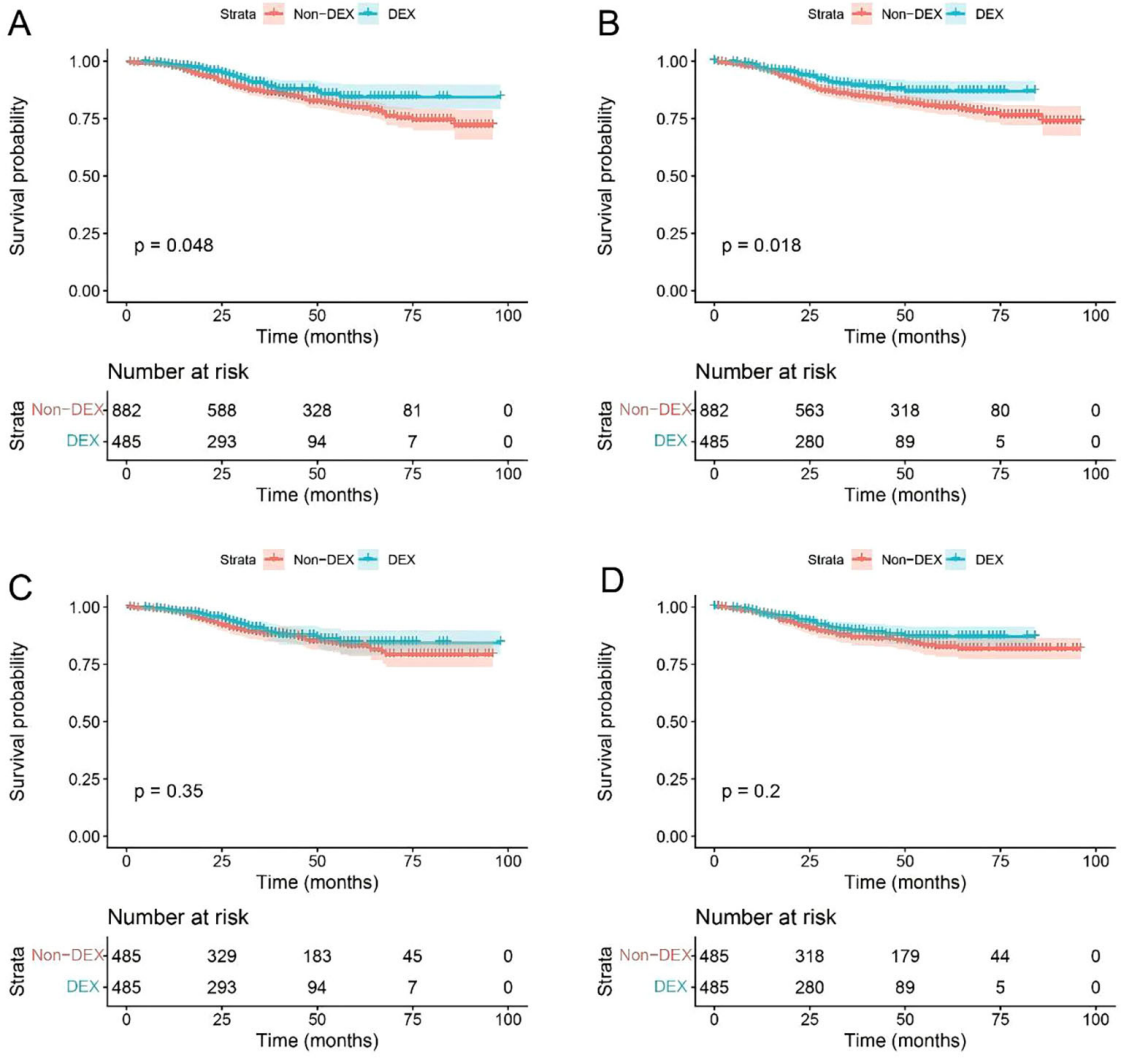
Survival curve of the patients before and after matching. **(A)** Overall survival before matching; **(B)** overall survival after matching; **(C)** disease-free survival before matching; **(D)** disease-free survival after matching. DEX, dexmedetomidine.

The Kaplan–Meier survival curve demonstrated a significantly higher overall survival probability for patients treated with DEX compared to those without this medication (*P* = 0.048 before matching, *P* = 0.018 after matching). However, no significant difference was found in disease-free survival probability (*P* = 0.350 before matching, *P* = 0.200 after matching) (**Figure 2**).

We performed dose-reactive relationship analyses for DEX on the main outcomes. The results showed that there was no significant dose dependence for all-cause death and recurrence or death risks (**Supplementary Table S4**). In addition, subgroup analyses showed no significant interaction between DEX and subgroup factors (tumor location, tumor differentiation, pathologic TNM stage, surgical type, and any complication before the operation) (**Supplementary Table S5**).

### The associations of DEX with secondary outcomes

In the overall cohort, linear regression models revealed a significant reduction in total hospital stay [adjusted *β* and 95% CI: −0.85 (−1.49, −0.22)] and postoperative hospital stay [adjusted *β* and 95% CI: −0.98 (−1.44, −0.52)] among patients in the DEX group. These findings remained statistically significant in the matched cohort [adjusted *β* and 95% CI for total hospital stay: −0.96 (−1.71, −0.22), adjusted *β* and 95% CI for postoperative hospital stay: −0.95 (−1.47, −0.43)]. However, no significant association was observed between DEX use in postoperative complications (**Table 4**).

**Table 4:** The associations of DEX with secondary outcomes.

Secondary outcomes	Unmatched		Matched
	Model 1	Model 2	Model 3
Total hospital stay, days^a^	−1.00 (−1.66, −0.35)	−0.85 (−1.49, −0.22)	−0.96 (−1.71, −0.22)
Postoperative hospital stay, days^a^	−1.04 (−1.49, −0.58)	−0.98 (−1.44, −0.52)	−0.95 (−1.47, −0.43)
Postoperative complication^b^	0.94 (0.64, 1.39)	1.01 (0.68, 1.50)	1.03 (0.66, 1.61)

Model 1: unadjusted in the overall cohort; model 2: adjusted for age, sex, BMI, TNM stage, preoperative ALB, preoperative HGB, preoperative CEA, differentiation degree, and history of abdominal operation in the overall cohort; model 3: the matched cohort.

DEX, dexmedetomidine.

a Multiple linear regression, *β*, and 95% CIs were displayed.

b Binary logistic regression, OR, and 95% CIs were displayed.

## Discussion

Our results showed that, among patients who underwent colorectal cancer surgery, intraoperative use of DEX was associated with improved overall survival, but not with improved disease-free survival. Patients receiving DEX had shorter total hospital stays and postoperative hospital stays. Furthermore, intraoperative use of DEX was not related to an increased risk of postoperative complications.

The impact of DEX on cancer outcomes is a fascinating topic. Preclinical investigations reported conflicting results. On the one hand, DEX could trigger tumorigenic signaling pathways by regulating specific enzymes and apoptotic molecules, thereby promoting tumor proliferation and migration (9, 17, 18). On the other hand, DEX inhibits the growth of lung adenocarcinoma cells and induces apoptosis by upregulating the expression of miR-493-5p (12). Furthermore, DEX inhibits the growth and metastasis of esophageal carcinoma cells through upregulating miR-143-3p and reducing epidermal growth factor receptor (13). These inconsistent findings may be attributed to DEX acting on different research subjects and activating different molecular mechanisms and signaling pathways.

Clinical studies investigating the long-term effects of DEX in patients with colorectal cancer are limited. In a follow-up analysis of patients with laparoscopic resection of colorectal cancer, DEX did not improve overall survival and disease-free survival but presented an increasing trend in overall survival and disease-free survival, indicating that the use of DEX may benefit the long-term prognosis of patients undergoing laparoscopic resection of colorectal cancer (19). In our study, the intraoperative administration of DEX was associated with improved overall survival, but not associated with disease-free survival (only marginal significance), which is a bit different from the results of the aforementioned study. The cause of this result may be that these were two different types of studies, with different baseline characteristics of the population, confounding factors, and inclusion and exclusion criteria. It is worth noting that patients receiving DEX had higher total intraoperative infusion volume even after propensity score matching, which may be another reason for the inconsistent results. We cannot control infusion volume as a confounder, because it appeared after using DEX, which violates the concept of confounding factors. Thus, a randomized controlled trial, a more effective method, may be needed to control the influence of intraoperative factors. Nevertheless, both studies showed an upward trend in overall survival and disease-free survival rates for patients who received DEX. In addition, we also explored the dose-reactive relationship between DEX and survival. We did not find that a higher dose of DEX can acquire a better survival. Given that this is a retrospective study, the accurate dose-reactive relationship needs to be validated in a future study.

Accumulating evidence suggests that DEX could mediate direct antitumor effects through various mechanisms, such as epigenetic modifications and the induction of apoptosis and ferroptosis (12, 13, 20, 21). In addition, DEX could alleviate stress response and inflammation, reduce the level of cortisol, and regulate immune function, thus inducing indirect antitumor effects (16, 22). Therefore, DEX can inhibit tumor growth in different ways. In a murine study, DEX showed inhibition of colorectal cancer growth by modulation of CD4^+^ and CD8^+^ T cells (23). These may indicate that intraoperative infusion of DEX may potentially inhibit tumor growth and improve clinical outcomes for colorectal cancer patients.

Our study controlled various potential confounding factors through propensity score matching, a method successfully applied in previous studies. However, this study has several limitations. First, residual confounding factors may still exist due to the retrospective property. Some covariates were difficult to collect, such as anesthetic drug dosages, depth of anesthesia, and intraoperative hemodynamic fluctuations. Second, we did not take into account the use of other anesthetics, hemodynamics, vasoactive drugs, and depth of anesthesia, resulting in failure to explain the intraoperative anesthetic-related environment. Third, retrospective studies cannot eliminate information bias, as electronic medical records may have some recording biases. Fourth, retrospective studies cannot elucidate the mechanisms that need to be explored through a basic study. Fifth, a further multicenter prospective study is necessary to validate the causal relationship.

In conclusion, this study demonstrated that intraoperative infusion of DEX is associated with decreased risk of all-cause mortality in patients who underwent colorectal cancer surgery. Patients who received DEX infusion exhibited shorter total hospital stays and postoperative hospital stays. These findings suggest that DEX infusion is a safe and feasible sedation scheme for patients undergoing colorectal cancer surgery.

## Data Availability

The original contributions presented in the study are included in the article/**Supplementary Material**. Further inquiries can be directed to the corresponding authors.
