# Supplementary material for: Association between intraoperative dexmedetomidine and survival outcomes after colorectal cancer surgery: a retrospective cohort study
Source: Front Oncol. 2025 Dec 2;15:1693496. doi: 10.3389/fonc.2025.1693496 (PMC12705362; doi:10.3389/fonc.2025.1693496)
Supplement: Supplementary file 1 [file DataSheet1.docx]

# **Association between Perioperative Dexmedetomidine and Survival Outcomes after Colorectal Cancer Surgery: A Retrospective Cohort Study**

Supplementary files

**Table S1** Proportional hazards (PH) assumption of Cox proportional hazards regression

**Table S2** Residual independence and collinearity diagnosis for multiple linear regression of total hospital stay

**Table S3** Residual independence and collinearity diagnosis for multiple linear regression of postoperative hospital stay

**Table S4** The dose-reactive relationship of DEX with main outcomes

**Table S5** Subgroup analyses of DEX with main outcomes

**Figure S1** Distribution of propensity score

**Figure S2** Bar chart of propensity score

**Figure S3** Love-plot before and after matching

**Figure S4** Residual normality for multiple linear regression of total hospital stay

**Figure S5** Homogeneity of residual variance and linear relationship of DEX and total hospital stay

**Figure S6** Residual normality for multiple linear regression of postoperative hospital stay

**Figure S7** Homogeneity of residual variance and linear relationship of DEX and postoperative hospital stay

**Table S1** Proportional hazards (PH) assumption of Cox proportional hazards regression

| Variables | OS | | | | |  | DFS | | | | |
| --- | --- | --- | --- | --- | --- | --- | --- | --- | --- | --- | --- |
|  | Unmatched | |  | Matched | |  | Unmatched | |  | Matched | |
|  | chisq | *P* |  | chisq | *P* |  | chisq | *P* |  | chisq | *P* |
| DEX | 0.183 | 0.669 |  | 0.0003 | 0.990 |  | 0.082 | 0.774 |  | 0.076 | 0.780 |
| Age | 2.924 | 0.087 |  |  |  |  | 0.066 | 0.798 |  |  |  |
| Sex | 2.552 | 0.110 |  |  |  |  | 1.602 | 0.206 |  |  |  |
| BMI | 0.829 | 0.363 |  |  |  |  | 0.026 | 0.873 |  |  |  |
| Preoperative HGB | 2.448 | 0.118 |  |  |  |  | 2.541 | 0.111 |  |  |  |
| Preoperative ALB | 0.811 | 0.368 |  |  |  |  | 0.973 | 0.324 |  |  |  |
| ASA | 0.207 | 0.902 |  |  |  |  | 0.641 | 0.423 |  |  |  |
| Comorbidity | 3.835 | 0.070 |  |  |  |  | 2.597 | 0.107 |  |  |  |
| Abdominal surgery | 0.009 | 0.924 |  |  |  |  | 1.260 | 0.262 |  |  |  |
| CEA | 1.530 | 0.216 |  |  |  |  | 0.371 | 0.543 |  |  |  |
| Tumor location | 5.940 | 0.051 |  |  |  |  | 3.769 | 0.052 |  |  |  |
| Tumor differentiation | 2.260 | 0.323 |  |  |  |  | 1.803 | 0.209 |  |  |  |
| Pathologic TNM stage | 1.486 | 0.476 |  |  |  |  | 1.887 | 0.204 |  |  |  |
| Surgical type | 0.258 | 0.612 |  |  |  |  | 0.994 | 0.319 |  |  |  |
| GLOBAL | 28.195 | 0.059 |  | 0.0003 | 0.990 |  | 2.839 | 0.102 |  | 0.076 | 0.780 |

DEX, dexmedetomidine; BMI, body mass index; HGB, hemoglobin; ALB, albumin; ASA, American Society of Anesthesiologists; CEA, carcinoembryonic antigen; TNM, tumor nodes metastasis; ICU, intensive care unit.

**Table S2** Residual independence and collinearity diagnosis for multiple linear regression of total hospital stay

|  | Unmatched | |  | Matched |
| --- | --- | --- | --- | --- |
|  | Model 1 | Model 2 |  | Model 3 |
| Durbin-Watson | 1.998 | 1.988 |  | 1.976 |
| Collinearity Statistics |  |  |  |  |
| Tolerance | 1 | 0.986 |  | 1 |
| VIF | 1 | 1.014 |  | 1 |

Model 1: Unadjusted in overall cohort; Model 2: Adjusted for age, sex, BMI, TNM stage, preoperative ALB, preoperative HGB, preoperative CEA, differentiation degree, history of abdominal operation, and preoperative comorbidity in overall cohort; Model 3: In matched cohort. VIF, variance inflation factor.

**Table S3** Residual independence and collinearity diagnosis for multiple linear regression of postoperative hospital stay

|  | Unmatched | |  | Matched |
| --- | --- | --- | --- | --- |
|  | Model 1 | Model 2 |  | Model 3 |
| Durbin-Watson | 1.918 | 1.909 |  | 1.975 |
| Collinearity Statistics |  |  |  |  |
| Tolerance | 1 | 0.986 |  | 1 |
| VIF | 1 | 1.014 |  | 1 |

Model 1: Unadjusted in overall cohort; Model 2: Adjusted for age, sex, BMI, TNM stage, preoperative ALB, preoperative HGB, preoperative CEA, differentiation degree, history of abdominal operation, and preoperative comorbidity in overall cohort; Model 3: In matched cohort. VIF, variance inflation factor.

**Table S4 The dose-reactive relationship of DEX with main outcomes**

|  | Unmatched [HR (95% CI)] | |  | Matched [HR (95% CI)] |
| --- | --- | --- | --- | --- |
|  | Model 1 | Model 2 |  | Model 4 |
| All–cause death |  |  |  |  |
| Non-DEX | 1 | 1 |  | 1 |
| DEX < 50 | 0.55 (0.30-0.99) | 0.59 (0.32-1.06) |  | 0.48 (0.27-0.83) |
| DEX 50-99 | 0.78 (0.45-1.35) | 0.78 (0.45-1.37) |  | 1.04 (0.55-1.98) |
| DEX ≥100 | 0.81 (0.46-1.44) | 0.84 (0.47-1.49) |  | 0.79 (0.44-1.44) |
| Recurrence or death |  |  |  |  |
| Non-DEX | 1 | 1 |  | 1 |
| DEX < 50 | 0.65 (0.41-1.01) | 0.70 (0.44-1.09) |  | 0.50 (0.33-0.77) |
| DEX 50-99 | 0.88 (0.58-1.36) | 0.85 (0.55-1.30) |  | 1.14 (0.70-1.85) |
| DEX ≥100 | 1.10 (0.73-1.65) | 1.11 (0.74-1.67) |  | 0.97 (0.64-1.48) |

Model 1: Unadjusted in overall cohort; Model 2: Adjusted for age, sex, BMI, TNM stage, preoperative ALB, preoperative HGB, preoperative CEA, differentiation degree, history of abdominal operation, and preoperative comorbidity in overall cohort; Model 3: In matched cohort.

**Table S5 Subgroup analyses of DEX with main outcomes**

| Subgroups | All–cause death [HR (95% CI)] | | | *P* for internation |  | Recurrence or death [HR (95% CI)] | | | *P* for internation |
| --- | --- | --- | --- | --- | --- | --- | --- | --- | --- |
|  | Incidence,  n (%) | Model 1 | Model 2 |  |  | Incidence,  n (%) | Model 1 | Model 2 |  |
| **Tumor location** |  |  |  | 0.358 |  |  |  |  | 0.214 |
| **Right colon** |  |  |  |  |  |  |  |  |  |
| Non-DEX | 32 (15.0) | 1 | 1 |  |  | 47 (22.0) | 1 | 1 |  |
| DEX | 12 (10.2) | 0.80 (0.41-1.55) | 0.84 (0.43-1.65) |  |  | 21 (17.8) | 0.95 (0.56-1.59) | 0.98 (0.58-1.66) |  |
| **Left colon** |  |  |  |  |  |  |  |  |  |
| Non-DEX | 40 (12.9) | 1 | 1 |  |  | 64 (20.6) | 1 | 1 |  |
| DEX | 14 (7.7) | 0.81 (0.44-1.49) | 0.80 (0.43-3.43) |  |  | 28 (15.4) | 1.08 (0.68-1.69) | 1.07 (0.68-1.69) |  |
| **Rectum** |  |  |  |  |  |  |  |  |  |
| Non-DEX | 59 (16.5) | 1 | 1 |  |  | 88 (24.6) | 1 | 1 |  |
| DEX | 13 (7.0)* | **0.56 (0.31-1.03)** | 0.71 (0.38-1.30) |  |  | 23 (12.4)* | 0.65 (0.41-1.04) | 0.74 (0.46-1.19) |  |
| **Tumor differentiation** |  |  |  | 0.195 |  |  |  |  | 0.359 |
| **Low** |  |  |  |  |  |  |  |  |  |
| Non-DEX | 8 (7.0) | 1 | 1 |  |  | 13 (11.4) | 1 | 1 |  |
| DEX | 4 (5.9) | 1.06 (0.32-3.55) | 2.20 (0.58-8.28) |  |  | 4 (5.9) | 0.80 (0.25-2.53) | 1.62 (0.47-5.58) |  |
| **Moderate** |  |  |  |  |  |  |  |  |  |
| Non-DEX | 41 (10.6) | 1 | 1 |  |  | 61 (15.7) | 1 | 1 |  |
| DEX | 9 (4.2)* | 0.58 (0.28-1.20) | 0.57 (0.27-1.20) |  |  | 22 (10.3) | 0.95 (0.58-1.55) | 0.98 (0.59-1.62) |  |
| **High** |  |  |  |  |  |  |  |  |  |
| Non-DEX | 82 (21.6) | 1 | 1 |  |  | 125 (32.9) | 1 | 1 |  |
| DEX | 26 (12.8)* | 0.70 (0.45-1.10) | 0.70 (0.45-1.09) |  |  | 46 (22.7)* | 0.81 (0.58-1.14) | 1.15 (0.41-3.18) |  |
| **Pathologic TNM stage** |  |  |  | 0.772 |  |  |  |  | 0.867 |
| **0–I stage** |  |  |  |  |  |  |  |  |  |
| Non-DEX | 8 (7.0) | 1 | 1 |  |  | 13 (11.4) | 1 | 1 |  |
| DEX | 4 (5.9) | 1.06 (0.32-3.55) | 2.20 (0.58-8.28) |  |  | 4 (5.9) | 0.80 (0.25-2.53) | 1.62 (0.47-5.58) |  |
| **II stage** |  |  |  |  |  |  |  |  |  |
| Non-DEX | 41 (10.6) | 1 | 1 |  |  | 61 (15.7) | 1 | 1 |  |
| DEX | 9 (4.2)* | 0.58 (0.28-1.20) | 0.57 (0.27-1.20) |  |  | 22 (10.3) | 0.95 (0.58-1.55) | 0.98 (0.59-1.62) |  |
| **III stage** |  |  |  |  |  |  |  |  |  |
| Non-DEX | 82 (21.6) | 1 | 1 |  |  | 125 (32.9) | 1 | 1 |  |
| DEX | 26 (12.8)* | 0.70 (0.45-1.10) | **0.70 (0.45-1.09)** |  |  | 46 (22.7)* | 0.81 (0.58-1.14) | 0.80 (0.57-1.13) |  |
| **Surgical type** |  |  |  | 0.257 |  |  |  |  | 0.061 |
| **Open surgery** |  |  |  |  |  |  |  |  |  |
| Non-DEX | 8 (16.7) | 1 | 1 |  |  | 13 (27.1) | 1 | 1 |  |
| DEX | 3 (16.7) | 1.82 (0.45-7.40) | 2.43 (0.54-11.20) |  |  | 6 (33.3) | 2.28 (0.93-6.26) | 2.29 (0.80-6.57) |  |
| **Laparoscopic surgery** |  |  |  |  |  |  |  |  |  |
| Non-DEX | 123 (14.7) | 1 | 1 |  |  | 186 (22.3) | 1 | 1 |  |
| DEX | 36 (7.7)* | **0.67 (0.46-0.97)** | **0.70 (0.48-1.02)** |  |  | 66 (14.1)* | **0.82 (0.61-1.08)** | 0.83 (0.63-1.11) |  |
| **Preoperative comorbidity** | |  |  | 0.53 |  |  |  |  | 0.59 |
| **No** |  |  |  |  |  |  |  |  |  |
| Non-DEX | 86 (14.1) | 1 | 1 |  |  | 132 (21.7) | 1 | 1 |  |
| DEX | 24 (7.0)* | **0.65 (0.41-1.02)** | **0.68 (0.43-1.08)** |  |  | 51 (14.9)* | 0.91 (0.65-1.26) | 0.93 (0.66-1.29) |  |
| **Yes** |  |  |  |  |  |  |  |  |  |
| Non-DEX | 45 (16.4) | 1 | 1 |  |  | 67 (24.5) | 1 | 1 |  |
| DEX | 15 (10.5) | 0.79 (0.44-1.42) | 0.86 (0.47-1.56) |  |  | 21 (14.7)* | 0.76 (0.46-1.24) | 0.77 (0.46-1.26) |  |

Model 1: Unadjusted in overall cohort; Model 2: Adjusted for age, sex, BMI, preoperative ALB, preoperative HGB, preoperative CEA, history of abdominal operation.

**P* < 0.05.


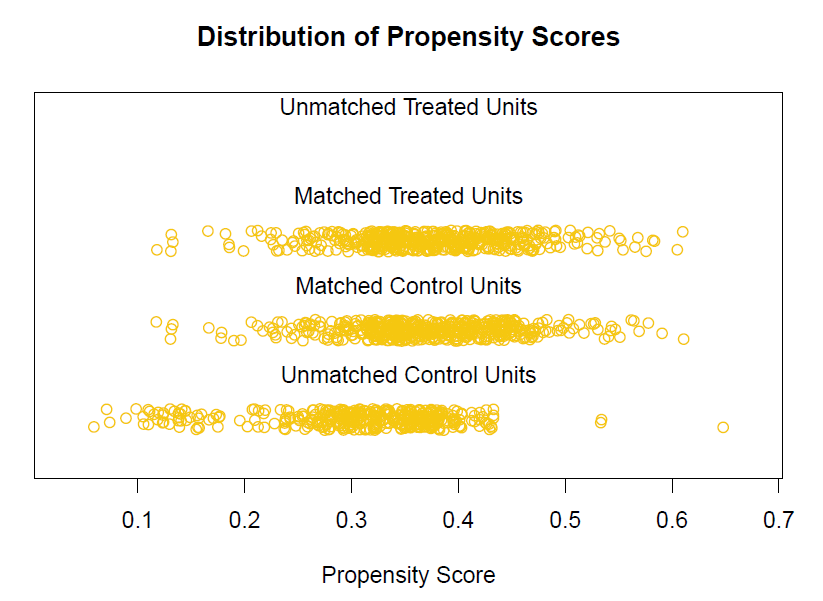


**Figure S1** Distribution of propensity score


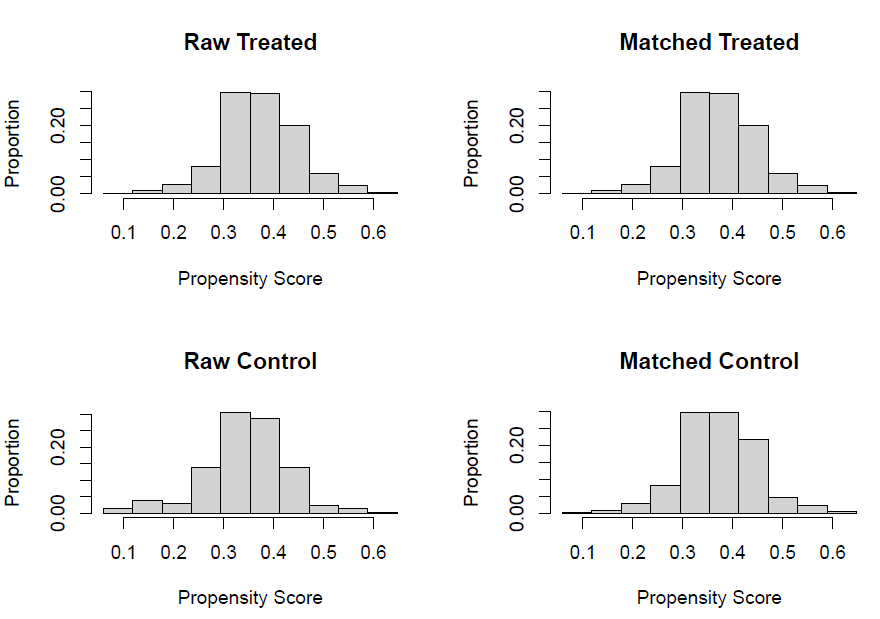


**Figure S2** Bar chart of propensity score


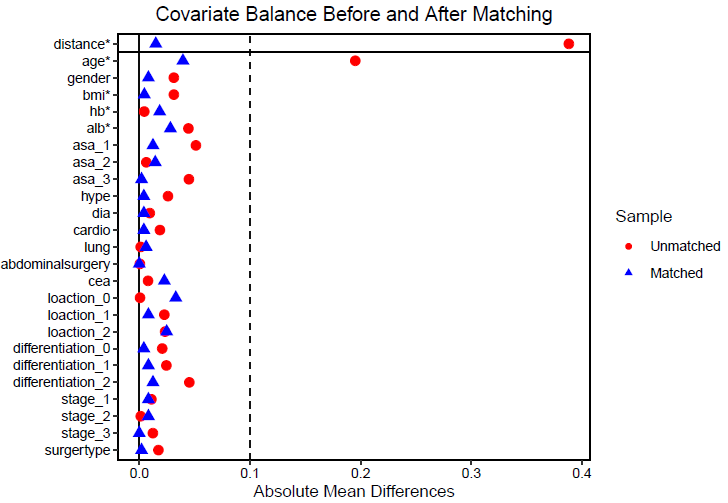


**Figure S3 Love-plot before and after matching**


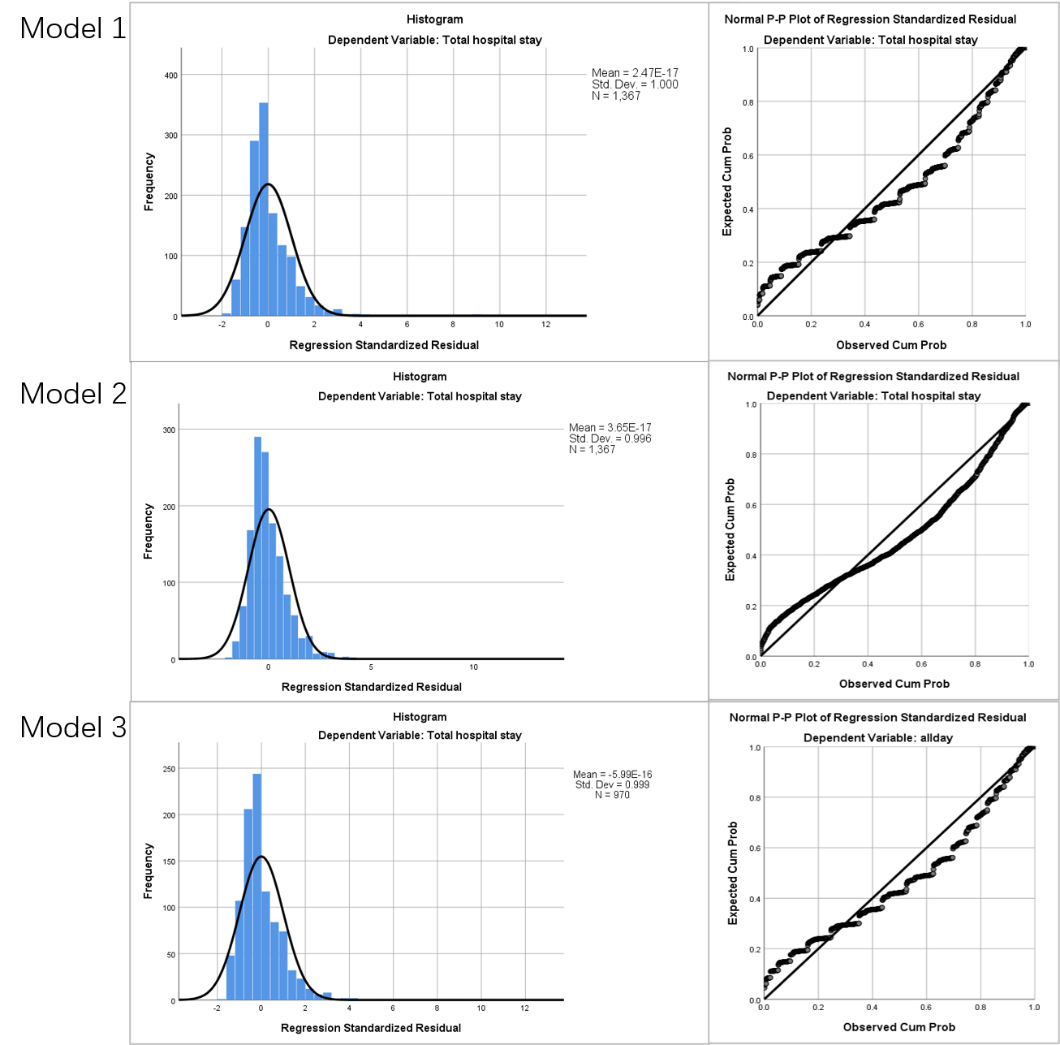


**Figure S4** Residual normality for multiple linear regression of total hospital stay


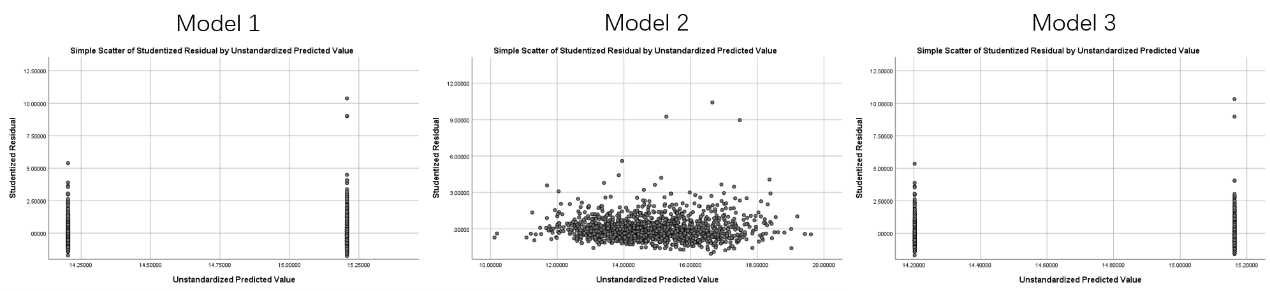


**Figure S5** Homogeneity of residual variance and linear relationship of dexmedetomidine and total hospital stay


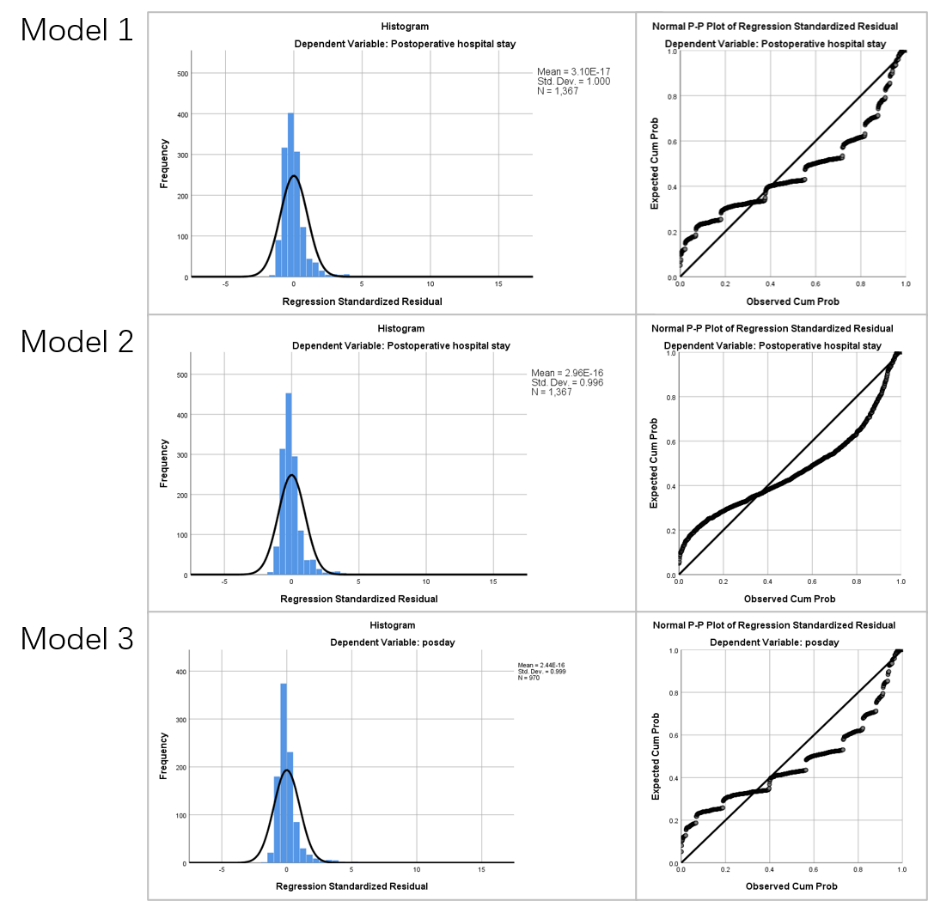


**Figure S6** Residual normality for multiple linear regression of postoperative hospital

Stay


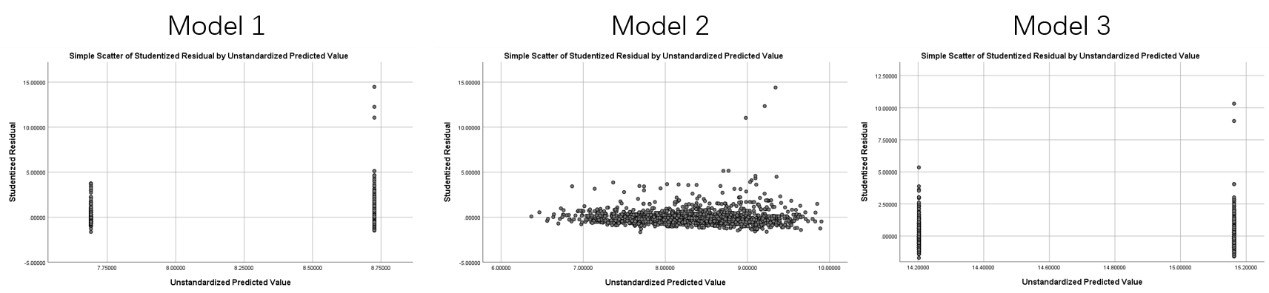


**Figure S7** Homogeneity of residual variance and linear relationship of dexmedetomidine and postoperative hospital stay
